# Drugs acting at TRPM7 channels inhibit seizure‐like activity

**DOI:** 10.1002/epi4.12773

**Published:** 2023-06-20

**Authors:** Aytakin Khalil, Tawfeeq Shekh‐Ahmad, Stjepana Kovac, Robert C. Wykes, F. David Horgen, Andrea Fleig, Matthew C. Walker

**Affiliations:** ^1^ Department of Clinical and Experimental Epilepsy UCL Queen Square Institute of Neurology, University College London London UK; ^2^ The Institute for Drug Research, The School of Pharmacy, Faculty of Medicine The Hebrew University of Jerusalem Jerusalem Israel; ^3^ Department of Neurology University of Münster Münster Germany; ^4^ Nanomedicine Lab, Division of Neuroscience University of Manchester Manchester UK; ^5^ Department of Natural Sciences Hawaii Pacific University Kaneohe Hawaii USA; ^6^ The Queen's Medical Center and John A. Burns School of Medicine and Cancer Center University of Hawaii Honolulu Hawaii USA

**Keywords:** carvacrol, epilepsy, seizure, TRPM7, waixenicin A

## Abstract

Transient receptor potential cation subfamily M7 (TRPM7) channels are ion channels permeable to divalent cations. They are abundantly expressed with particularly high expression in the brain. Previous studies have highlighted the importance of TRPM7 channels in brain diseases such as stroke and traumatic brain injury, yet evidence for a role in seizures and epilepsy is lacking. Here, we show that carvacrol, a food additive that inhibits TRPM7 channels, and waixenicin A, a novel selective and potent TRPM7 inhibitor, completely suppressed seizure‐like activity in rodent hippocampal‐entorhinal brain slices exposed to pentylenetetrazole or low magnesium. These findings support inhibition of TRPM7 channels as a novel target for antiseizure medications.

## 1 INTRODUCTION

Transient receptor potential cation subfamily M7 (TRPM7) channels are ubiquitously expressed and constitutively active transmembrane ion channels that are abundantly expressed in the brain.^1^, ^2^ TRPM7 channels are suppressed by intracellular magnesium and are activated when magnesium is depleted.^3^ TRPM7 channels are permeable to divalent ions including Ca^2+^ and can depolarize neurons. Importantly, conditions associated with low serum magnesium levels can lead to seizures,^4^, ^5^ thus possibly linking the TRPM7 channel with epilepsy.

Besides low levels of magnesium, low extracellular Ca^2+^ concentrations, reactive oxygen species, and decreased pH, which have also all been strongly linked to seizure activity, can activate TRPM7 channels.^1^ Despite this, the role of TRPM7 channels in seizures and/or epilepsy is still unclear.

Carvacrol (Figure S1), a food additive, is a TRPM7 inhibitor, which has been demonstrated to have antiseizure effects in vivo,^2^, ^6^, ^7^ although the mechanisms underlying this are unclear. We, therefore, asked whether carvacrol has a direct antiseizure effect, indicating that TRPM7 inhibition can have an antiseizure effect. However, carvacrol has been suggested to have other targets, including sodium channels and GABA(A) receptors.^8^, ^9^ To provide further support for a role of TRPM7 in generating seizure activity, we tested waixenicin A (Figure S1), a highly selective and potent TRPM7 channel inhibitor that permits selective targeting of TRPM7 channels.^10^ We show here that these two TRPM7 channel blockers suppress epileptiform activity in vitro, providing strong evidence that targeting TRPM7 is a novel approach for seizure control.

## 2 METHODS

### 2.1 Animals

All animal procedures were carried out subject to local ethical approval and followed the UK Home Office Animal (Scientific Procedures) Act, 1986. Male Sprague‐Dawley rats (3–3.5 weeks old, Charles River) were housed under controlled conditions; at a temperature of 22°C, maintained on a 12/12 h dark/light cycle with free access to food and water. All animal procedures were carried out with local ethical approval and adhered to the UK Home Office Animal (Scientific Procedures) Act, 1986.

### 2.2 Preparing acute brain slices

All compounds were purchased from Sigma Aldrich, UK unless stated differently. For acute brain slices, rats were culled with isoflurane overdose and then decapitated. Horizontal brain slices comprising the hippocampus, dentate gyrus, and entorhinal cortex were prepared on the vibratome (Leica VT1200S, Leica Microsystems). All solutions were enriched with 95% O_2_/5%CO_2_ gas to maintain pH 7.4–7.35 and to provide sufficient tissue oxygenation. Slices were kept in artificial cerebrospinal fluid (ACSF) containing 126 mM NaCl, 2.5 mM KCl, 0.9 mM NaH_2_PO_4_, 14 mM D‐glucose, 2.5 mM CaCl_2_, 1 mM MgCl_2,_ and 2.6 mM NaHCO_3_: osmolality 296 mOsm/kg. For recordings, slices were transferred to low Mg^2+^ ACSF solution, in which MgCl_2_ was omitted and the KCl concentration was increased to 5 mM. Epileptiform activity in the pentylenetetrazole (PTZ) model of epilepsy was elicited by adding 2 mM of PTZ to high potassium (5 mM) ACSF solution. The submersion recording chamber was continuously perfused with solution enriched with 95%O_2_/5%CO_2_ at ~4 mL/min and temperature was controlled at ~33°C. Low Mg^2+^ or PTZ‐induced epileptiform discharges were recorded from CA1 region of the hippocampus. The recording electrode was filled with the perfusion solution. Recordings were obtained using a Multi‐Clamp 700B or Axopatch 200B amplifier (Molecular Devices), and were low‐pass filtered at 4 kHz. WinEDR (Strathclyde Electrophysiology Software) was used for data acquisition. Sampling rate was 10 kHz. Two TRPM7 channel blockers were used: *carvacrol* (Sigma Aldrich) and *waixenicin A* (kindly provided by DH from Hawaii Pacific University).^10^

Carvacrol is an oil and so we initially compared carvacrol (1 mM) dissolved in DMSO (0.26%) prior to adding it to the perfusion solution in seven experiments and in the other seven experiments carvacrol was added without DMSO. In six control experiments DMSO was added as a vehicle to the perfusion solution (0.26%) after 20 min of the baseline recording to control for a potential effect of DMSO on epileptiform activity. There was no significant difference between these two sets of experiments (DMSO and carvacrol [1 mM] and carvacrol [1 mM] alone) and there was no effect of DMSO at this concentration on epileptiform activity in control conditions. Thus, experiments with and without DMSO were pooled for the final analysis. Based on these results lower concentrations of carvacrol (50–800 μM) were added without DMSO.

To block TRPM7 channel 10–20 μM of waixenicin A was used in the low Mg^2+^ experiments, and 10 μM (minimum effective dose) in the PTZ experiments. The specific protocol provided by DH from Hawaii Pacific University was used to dissolve waixenicin A. In brief, 50 μL of 99% methanol was added to the vial containing 50 μg waixenicin A, and then once half of methanol volume evaporated 1035 μL of a buffer (PTZ or low Mg^2+^ containing ACSF) was added to dissolve the compound completely. The final concentration of methanol in the perfusion solution was 0.23% in PTZ and 0.23%–0.46% in low Mg^2+^ experiments: these concentrations of methanol were added in control experiments with PTZ and low Mg^2+^.

### 2.3 Epileptiform activity

Epileptiform bursts were defined as field potential activity with an amplitude more than three times that of baseline, containing more than two subsequent spikes that do not return to baseline, and with overall duration ≥40 ms. Random short single spikes or polyspikes that were distinct from epileptiform activity recorded during baseline, and which did not meet the above criteria were observed in all experiments. These were not included in the count of burst activity (Figure S2). Recordings with both ictal‐like and interictal‐like activity were included in the analyses in the low Mg^2+^ in vitro model to test the effect of the TRPM7 blocker.

### 2.4 Data analyses and statistics

All in vitro recordings were obtained from WinEDR 3.1.9. (Strathclyde Electrophysiology Software) and were analyzed using pCLAMP 10.3 software (Molecular Devices). Each event was selected manually after applying a threshold detection tool. Unpaired *t* tests were used to analyze the frequency of epileptiform activity prior testing for Gaussian distribution (*P* > 0.05, Shapiro‐Wilk test). Statistical analyses were carried out using STATA V.11 (StataCorp). The threshold for significance was set at *P* < 0.05.

## 3 RESULTS

**FIGURE 1:**
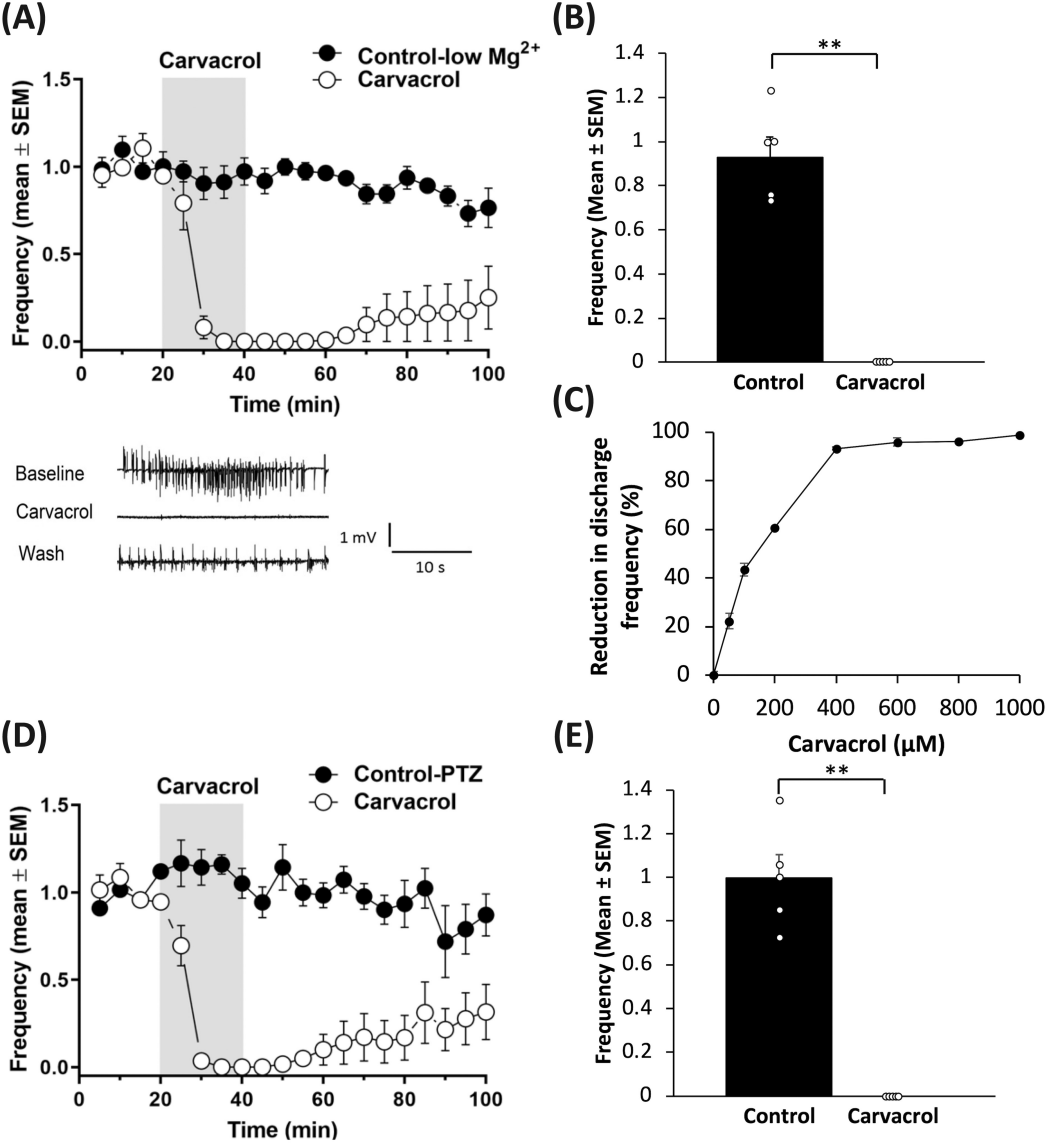
Carvacrol inhibits low magnesium and pentylenetetrazole (PTZ)‐induced epileptiform activity in vitro. A, Graph of mean (±SEM) frequency of low magnesium‐induced epileptiform discharges over time in slices exposed to carvacrol (*n* = 5) or vehicle (*n* = 5). B, Summary bar chart of (A) for effect on epileptiform discharges 20 min after carvacrol/vehicle application. C, Dose‐response curve for the effect of carvacrol on low magnesium‐induced epileptiform activity (*n* = 4–6 for each point). D, Graph of mean (±SEM) frequency of PTZ‐induced epileptiform discharges over time in slices exposed to carvacrol (*n* = 5) or vehicle (*n* = 5). E, Summary bar chart of (D) for effect on epileptiform discharges, 20 min after carvacrol/vehicle application. **P < 0.01.

We first tested the effect of the TRPM7 channel blocker carvacrol on low magnesium‐induced seizure‐like activity. Carvacrol (1 mM) significantly inhibited the frequency of low Mg^2+^‐induced epileptiform activity compared to control (*P* < 0.001, *n* = 5 for each group, two‐sample *t* test; Figure 1A,B). Five minutes after carvacrol administration epileptiform activity started to decrease and was completely abolished after 10 min. There was only partial washout (Figure 1A). In contrast, the frequency of epileptiform activity in control recordings, performed over the same period (100 min), was constant in the initial 80 min with a small decrease in some recordings thereafter. We next determined the concentration‐response relationship for carvacrol on low magnesium‐induced epileptiform activity in vitro. Carvacrol suppressed epileptiform activity with an EC50 of 170.4 ± 33.6 μM (Figure 1C).

It is known that TRPM7 channels are activated by low extracellular magnesium.^11^ To exclude that the effect of carvacrol on in vitro epileptiform activity was mainly due to the model used, that is, the low magnesium model of seizure‐like activity, and to test whether TRPM7 channels would be activated during seizure‐like activity in normal Mg^2+^ concentrations, we used another in vitro model in which epileptiform activity is induced in physiological extracellular magnesium concentrations. PTZ, a GABA(A) receptor antagonist, at 2 mM induced epileptiform events in combined hippocampal–entorhinal cortex slices after ~30–40 min of exposure.

We similarly observed a decrease in epileptiform activity within 5 min in the PTZ model in brain slices treated with carvacrol. The activity was completely abolished within 10 min. Carvacrol significantly reduced the frequency of PTZ‐induced epileptiform discharges (two‐sample *t* test, *n* = 5 for each group, *P* < 0.001; Figure 1D,E). During the washout period no or a partial restoration of epileptiform activity was seen.

**FIGURE 2:**
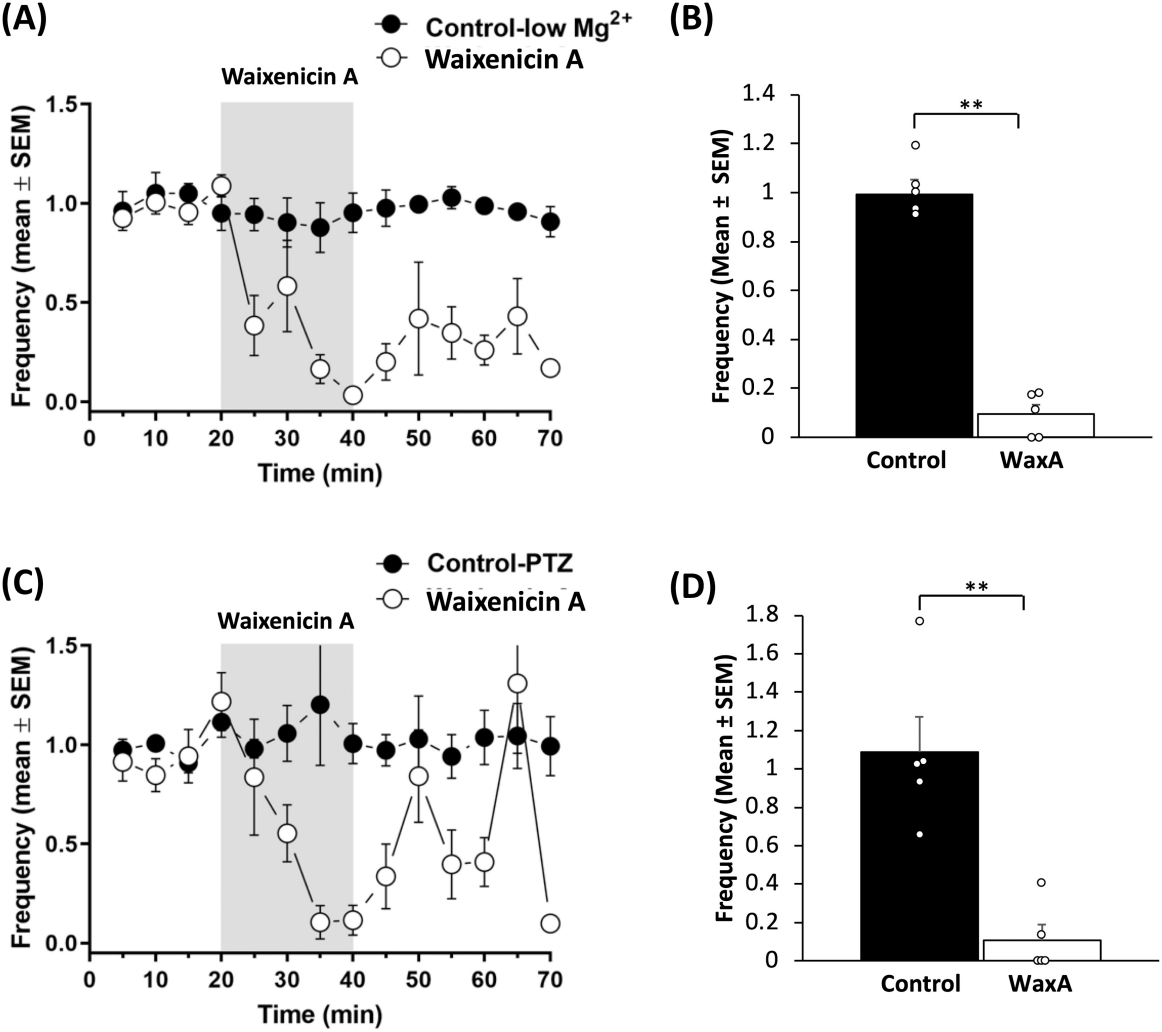
Waixenicin A inhibits low magnesium‐ and pentylenetetrazole (PTZ)‐induced epileptiform activity in vitro. Graphs of mean (±SEM) frequency of low magnesium‐induced (A) and PTZ‐induced (C) of epileptiform discharges over time. Note that TRPM7 blocker waixenicin A (10 μM) decreases in vitro epileptiform activity. Bar charts summarizing frequency of low magnesium‐ induced (B) and PTZ‐induced (D) epileptiform discharges, 20 min after waixenicin A application or vehicle application. *n* = 5 for each group. **P < 0.01.

To support that the effect of carvacrol on in vitro epileptiform activity was through inhibition of TRPM7 channels, we tested the effect of waixenicin A, a novel selective and potent inhibitor of TRPM7 channels, on in vitro epileptiform activity. Waixenicin A also reduced epileptiform activity in slices in the low Mg^2+^ model with a complete block of epileptiform discharges after 20 min of perfusion with waixenicin A. Blocking TRPM7 channels by waixenicin A significantly reduced the frequency of epileptiform discharges (*P* < 0.001, *n* = 5 in each group, two‐sample *t* test; Figure 2A,B). During washout of the drug, epileptiform activity was partially restored. A very similar pattern was seen in the PTZ model of epilepsy, in which waixenicin A significantly reduced the frequency of PTZ‐induced epileptiform discharges (*P* < 0.001, two‐sample *t* test, *n* = 5 in each group, Figure 2C,D).

## 4 DISCUSSION

We here show that carvacrol and waixenicin A significantly reduce in vitro epileptiform activity in two different models of epileptiform activity. Previous studies have confirmed a neuroprotective effect of TRPM7 channel blocker carvacrol in animal models of seizures, hypoxic‐ischemic injury and traumatic brain injury.^2^, ^11^, ^12^, ^13^ To our knowledge this is the first study that shows an effect of TRPM7 channel inhibition on in vitro epileptiform activity. The advantage of using these in vitro models is that they avoid confounders of in vivo pharmacokinetics and potential metabolites encountered with in vivo models. However, in vitro models do not completely recapitulate in vivo seizure activity, and so further in vivo studies will be necessary to confirm the impressive efficacy seen in vitro.

Carvacrol, an essential oil of oregano (*Origanum vulgare* L.) and thyme (*Thymus vulgaris* L.),^14^ has multiple targets. Carvacrol has a stimulatory effect on thermo‐TRP channels, TRPV3 and TRPA1,^15^ which is unlikely to contribute to its antiseizure effect. Carvacrol also inhibits drosophila TRPL channel, which is a mammalian TRPC analogue^16^; TRPC channels have been implicated in ictogenesis and epileptogenesis.^17^ Two studies suggested inhibition of peripheral sodium channels by carvacrol,^9^, ^18^ and carvacrol has been reported to activate GABA(A) receptors and potentiate the effects of benzodiazepines.^8^ In contrast, we have shown that carvacrol had no effect on the fiber volley or paired‐pulse inhibition in hippocampal slices, indicating that it does not have a significant inhibitory effect on sodium channels or GABA(A) receptors in the system under study here.^2^ The effect of carvacrol was not completely reversible during washout period in both low Mg^2+^ and PTZ experiments, which is likely due to nonspecific binding of carvacrol in the slice preparation.

Since carvacrol has off‐target effects, we also tested waixenicin A, derived from the soft coral *Sarcothelia edmondsoni*. Waixenicin A has been identified as a strong and specific inhibitor of overexpressed and native TRPM7 channels.^10^ In our experiments waixenicin A significantly decreased the frequency of epileptiform activity. The concentrations used are similar to the concentrations used in previous studies. Since the initial description by Zierler and colleagues, waixenicin A has been used as a specific TRPM7 channel blocker to study the effect of TRPM7 channels in the context of cancer^19^, ^20^ or in the context of axonal outgrowth.^21^ Here, for the first time, we have demonstrated an effect of waixenicin A in models of epileptiform activity. TRPM7 channels are thus potential and effective and novel targets for antiseizure medication development.

## CONFLICT OF INTEREST STATEMENT

None of the authors has any conflict of interest to disclose. We confirm that we have read the Journal's position on issues involved in ethical publication and affirm that this report is consistent with those guidelines.

## Supporting information


Figure S1:
Figure S2:
