# Supplementary material for: Drugs acting at TRPM7 channels inhibit seizure‐like activity
Source: Epilepsia Open. 2023 Jun 20;8(3):1169–74. doi: 10.1002/epi4.12773 (PMC10472385; doi:10.1002/epi4.12773)
Supplement: Supplementary file 1 — Figure S1: Figure S2: [file EPI4-8-1169-s001.docx]

**Supplementary Figures**

**Suppl. Fig. 1:** Chemical structure of carvacrol and waixenicin A^12^.

**Suppl. Fig. 2:** Examples of burst like activity in the low magnesium model (A), in the PTZ model (B) of seizure-like activity and examples of random short single spikes (C). Note that activity in A and B was included in the count of burst activity whereas activity in C was not counted.
